# Sex-Related Differences in the Immune Response to Meningococcal Vaccinations During Adolescence

**DOI:** 10.3389/fpubh.2022.871670

**Published:** 2022-05-06

**Authors:** Milou Ohm, Anna G. C. Boef, Susanne P. Stoof, Mariëtte B. van Ravenhorst, Fiona R. M. van der Klis, Guy A. M. Berbers, Mirjam J. Knol

**Affiliations:** Centre for Infectious Disease Control, National Institute for Public Health and the Environment (RIVM), Bilthoven, Netherlands

**Keywords:** *Neisseria meningitidis*, sex differences, vaccine response, antibody levels, meningococcal vaccination, adolescents

## Abstract

**Background:**

Immune responses to pediatric vaccinations have been reported to differ according to sex. Such sex-differential responses may become more pronounced during adolescence due to hormonal differences. We investigated whether the vaccine response following primary vaccination against meningococcal serogroup A (MenA), MenW and MenY and booster vaccination against MenC differed between girls and boys using data from two clinical studies.

**Methods:**

Children aged 10, 12, and 15 years, who had been primed with MenC vaccination between 14 months and 6 years of age, received a booster MenC vaccination or MenACWY vaccination. Polysaccharide-specific IgG concentrations and functional antibody titers [determined with the serum bactericidal antibody (SBA) assay] were measured at baseline, 1 month, 1 year, and 3 years (only MenC group) after vaccination. We calculated geometric mean concentrations and titers (GMC and GMT) ratios for girls vs. boys adjusted for age group. Additionally, we compared the proportion protected individuals between girls and boys at all timepoints.

**Results:**

This study included 342 girls and 327 boys from two clinical trials. While MenAWY antibody levels did not differ consistently 1 month after vaccination, all GMC- and GMT-ratios were in favor of girls 1 year after vaccination [range: 1.31 (1.02–1.70) for MenA IgG to 1.54 (1.10–2.16) for MenW IgG]. Overall, MenC antibody levels were slightly higher in girls at all postvaccination timepoints (GMC- and GMT-ratios: 1.16/1.17 at 1 month, 1.16/1.22 at 1 year and 1.12/1.15 3 years postvaccination). Higher MenC antibody levels were observed in 12- and 15-year-old girls compared to boys of the same age, whereas 10-year-old boys and girls had similar antibody levels. The percentage of participants protected (SBA titer ≥ 8) was very high (95–100%) at all timepoints, and did not differ significantly between boys and girls.

**Conclusion:**

Antibody responses were higher in girls than in boys for all serogroups at most timepoints after primary MenAWY vaccination and booster MenC vaccination. The differences in average titers were however small and the percentage participants with protective titers was very high for both sexes.

## Introduction

Sex-related differences of genetic and hormonal nature are known to influence the immune system (1). Biological factors related to sex, such as hormones, but also chromosomal differences are considered important in both infectious diseases and autoimmunity (2). Invasive meningococcal disease (IMD) is a severe disease, caused by the Gram-negative bacterium *Neisseria meningitidis* (3), which can be prevented by vaccination. A meta-analytic evaluation of sex differences in IMD rates by age group in 10 countries found excess incidence rates in young males, but a reversed sex ratio in older adults with higher rates in females (4). During a recent IMD-W outbreak in the Netherlands, females were affected more often than males (66 vs. 34% respectively), although cases predominantly occurred in (older) adults (5). Mortality data from New York City showed higher case fatality rates for IMD in females across all ages (6). However, there is insufficient knowledge about the vaccine response at different stages of life in relation to sex and a paucity of clinical (vaccine) trials that include data analyzed by sex (7, 8). Immune responses to several infant vaccinations have been reported to differ according to sex (9). Such sex-differential responses may become more pronounced during adolescence due to hormonal differences. For example, while IgG and IgM levels are generally equal between the sexes pre-puberty, these immunoglobulins are higher in females post-puberty (2). Knowledge on sex differences in vaccine response could contribute to the rationale of vaccine strategies, as was previously proposed for influenza vaccination (10).

A meningococcal serogroup C (MenC) conjugate vaccine was introduced in the national immunization programme (NIP) in the Netherlands in September 2002 for 14-month-olds (11); children born from July 2001 onwards were therefore eligible for vaccination. Furthermore, a catch-up campaign for children up to 18 years of age (born from June 1983 until July 2001) was conducted from June until November 2002 (11). Recently, the MenC conjugate vaccine was replaced by a meningococcal serogroup A, C, W and Y (MenACWY) conjugate vaccine in response to an increase of IMD serogroup W (IMD-W) (12). During this increase, teenagers were the main target population for vaccination, since they were disproportionally affected during this increase (13) and since this age group has the highest meningococcal carriage rate (14). A mass campaign for 14–18 year-olds (born between January 2001 and December 2005) was conducted, and all 14-year-olds are now offered a MenACWY-TT booster dose, after priming at the age of 14 months. Data on protection levels after meningococcal vaccination separated by sex are scarce and lacking for adolescents in particular.

Our objective was to explore the sex-related differences in the immune response following adolescent meningococcal vaccination in two clinical studies that were carried out between 2011–14 and 2015–19. We determined the quantity and functionality of serum and salivary MenACWY antibody levels in individuals aged 10, 12 and 15 years at time of vaccination, and assessed differences between the sexes.

## Materials and Methods

### Study Populations

Two phase-IV clinical trials (clinical trial numbers: NL3372 and NL4286) enrolled participants in 2011 and 2014 to receive a MenC-TT or MenACWY-TT vaccine, respectively, at the age of 10, 12 or 15 years after being primed at young age (aged between 14 months and 6 years) with a MenC-TT vaccine, as previously described (15–17). Serum samples were collected at baseline (T0), 1 month (T1) and 1 year (T2) after vaccination. In addition, from a subset of participants serum samples were collected at 3 years (T3) postvaccination (MenC booster vaccination group) (18).

### Serological Analyses

MenA-, MenC-, MenW-, and MenY-PS-specific serum IgG, serum IgA and salivary IgA concentrations and tetanus toxoid (TT)-specific serum IgG concentrations were measured using a fluorescent-bead-based multiplex immunoassay (MIA) (19–22). Functional antibodies were assessed with the serum bactericidal antibody (rSBA) assay using baby rabbit complement and MenA strain 3125, MenC strain C11 (23), MenW strain MP01240070 and MenY strain S-1975 as target strains. The correlate of protection (internationally accepted) of rSBA titer ≥ 8 was used for analyses, with the bactericidal titer defined as the dilution of the serum that corresponded with ≥50% killing after 60 min incubation (24–26). When the titer fell below the cut-off of the assay (titer <4), a value of 2 was assigned.

### Statistical Analyses

The statistical analyses were performed using Excel, GraphPad Prism 8 and SPSS Statistics v24. Geometric mean concentrations (GMCs) of meningococcal serogroups A, C, W and Y polysaccharide (PS)-specific IgG and TT-specific IgG and geometric mean titers (GMTs) for serogroup-specific SBA titers were calculated for girls and boys separately (across age groups) at T1 (1 month after booster vaccination) and T2 (1 year after booster vaccination). We used a generalized linear model to perform regression analyses per serogroup, using ln-transformed IgG levels or SBA titer at T1 or T2 as dependent variable and sex as independent variable. The exponentiated regression coefficient for sex was used to obtain IgG GMC ratios or SBA GMT ratios for girls vs. boys for each serogroup. We performed the MenAWY analyses (1) adjusted for age group and (2) adjusted for both age group and IgG or SBA at T0. For meningococcal serogroup A, W and Y, we did not perform separate analyses for the different age groups because of the small sample sizes. We performed MenC analyses (1) adjusted for study-group (8 groups which differed on the following aspects: booster age, priming age and MenC-TT or MenACWY-TT booster vaccination) and (2) adjusted for both study-group and IgG or SBA result at T0. We performed analyses per booster-age-group (10, 12, and 15 years), and overall for each timepoint. Analyses were performed for an additional timepoint (T3: 3 years after MenC booster vaccination) for the subgroup for whom measurements at this additional timepoint were available. In addition, the proportion of protected (SBA titer ≥ 8) girls and boys at the different time points for each serogroup were compared by a Fischer's exact test. For serum IgA and salivary IgA we performed the same analyses as for IgG and SBA. No measurements at 3 years after booster vaccination were available for serum or salivary IgA. The same analyses were also performed for TT-specific serum IgG for the MenC booster vaccination group, with measurements available at baseline, 1 month and 1 year after vaccination. A *p*-value of <0.05 was considered as statistically significant.

## Results

### Population Characteristics

As shown in Table 1A, the distribution of girls and boys slightly differed across age groups in the study population for meningococcal serogroup A, W and Y with more girls in the youngest age group and more boys in the older age groups. Baseline IgG levels against meningococcal serogroups A, W and Y were generally low for both sexes. The percentage with protective SBA titers at baseline was similar for girls and boys for all three serogroups, with overall 20, 15, and 31% of the participants protected for serogroup A, W, and Y, respectively.

**Table 1A:** Characteristics of the study population for meningococcal serogroups A, W, and Y.

**Characteristic**	**Girls (*n* = 121)**	**Boys (*n* = 116)**
**Age group**, ***n*** **(%)**		
10 y	47 (38.8)	33 (28.4)
12 y	36 (29.8)	43 (37.1)
15 y	38 (31.4)	40 (34.5)
**Baseline IgG in** **μg/mL, median (IQR)**		
MenA	0.55 (0.29–1.39) *(n = 118)*	0.44 (0.25–0.85) *(n = 114)*
MenW	0.12 (0.05–0.44) *(n = 119)*	0.08 (0.04–0.23) *(n = 115)*
MenY	0.06 (0.03–0.12) *(n = 119)*	0.05 (0.03–0.08) *(n = 115)*
**Baseline SBA titer**		
MenA, median (range)	2 (2–2,048)	2 (2–1,024)
MenA ≥ 8, *n* (%)	24/117 (21)	22/115 (19)
MenW, median (range)	2 (2–512)	2 (2–2,048)
MenW ≥ 8, *n* (%)	18/118 (15)	18/115 (16)
MenY, median (range)	2 (2–4,096)	2 (2–4,096)
MenY ≥ 8, *n* (%)	35/117 (30)	38/115 (33)

*MenA, meningococcal serogroup A; MenW, meningococcal serogroup W135; MenY, meningococcal serogroup Y; IgG, immunoglobulin G; SBA, serum bactericidal antibody; IQR, interquartile range*.

The characteristics of the study population for meningococcal serogroup C are described in Table 1B. Both the baseline MenC IgG concentrations and the percentage with protective SBA titers at baseline did not differ between girls and boys. The overall percentage of participants with protective SBA titers at baseline ranged from 10% among 12-year olds who were primed at 14 months of age, to 45% in 15-year olds who were primed at 6 years of age.

**Table 1B:** Characteristics of the study population for meningococcal serogroups C.

**Characteristic**			**Girls (*n* = 342)**	**Boys (*n* = 327)**
Group^*^, ***n*** **(%)**				
10 y	MenC-TT	14 m	53 (15.5)	38 (11.6)
10 y	MenACWY-TT	14 m	47 (13.7)	33 (10.1)
12 y	MenC-TT	3 y	44 (12.9)	47 (14.4)
12 y	MenC-TT	14 m	37 (10.8)	45 (13.8)
12 y	MenACWY-TT	14 m	36 (10.5)	43 (13.2)
15 y	MenC-TT	6 y	41 (12.0)	45 (13.8)
15 y	MenC-TT	3 y	46 (13.5)	36 (11.0)
15 y	MenACWY-TT	3 y	38 (11.1)	40 (12.2)
**Baseline IgG in** **μg/mL, median (IQR)**			
**MenC**			
Overall		0.26 (0.15–0.51) *(n = 338)*	0.24 (0.14–0.46) *(n = 326)*
10 y	14 m	0.21 (0.12–0.43) *(n = 98)*	0.27 (0.13–0.53) *(n = 70)*
12 y	3 y	0.24 (0.15–0.66) *(n = 44)*	0.26 (0.18–0.47) *(n = 47)*
12 y	14 m	0.21 (0.10–0.43) *(n = 71)*	0.21 (0.11–0.43) *(n = 88)*
15 y	6 y	0.45 (0.28–0.83) *(n = 41)*	0.25 (0.16–0.52) *(n = 45)*
15 y	3 y	0.28 (0.18–0.51) *(n = 84)*	0.24 (0.14–0.46) *(n = 76)*
**Baseline SBA titer**			
**MenC, median (range)**			
Overall		2 (2–16,384)	2 (2–16,384)
10 y	14 m	2 (2–2,048)	2 (2–512)
12 y	3 y	2 (2–3,072)	2 (2–4,096)
12 y	14 m	2 (2–1,024)	2 (2–2,048)
15 y	6 y	4 (2–16,384)	2 (2–768)
15 y	3 y	2 (2–2,048)	2 (2–16,384)
**MenC** **≥** **8**, ***n*** **(%)**			
Overall		66/337 (19.6)	70/326 (21.5)
10 y	14 m	12/98 (12)	13/70 (19)
12 y	3 y	14/44 (32)	17/47 (36)
12 y	14 m	6/71 (9)	10/88 (11)
15 y	6 y	20/41 (49)	19/45 (42)
15 y	3 y	14/83 (17)	11/76 (15)

*MenC, meningococcal serogroup C; IgG, immunoglobulin G; SBA, serum bactericidal antibody; IQR, interquartile range.*

* *Groups differed on the following aspects: (1) booster age, (2) MenC-TT or MenACWY-TT booster vaccination, and (3) priming age*.

### Meningococcal Serogroups A, W and Y: IgG and SBA

**Figure 1:**
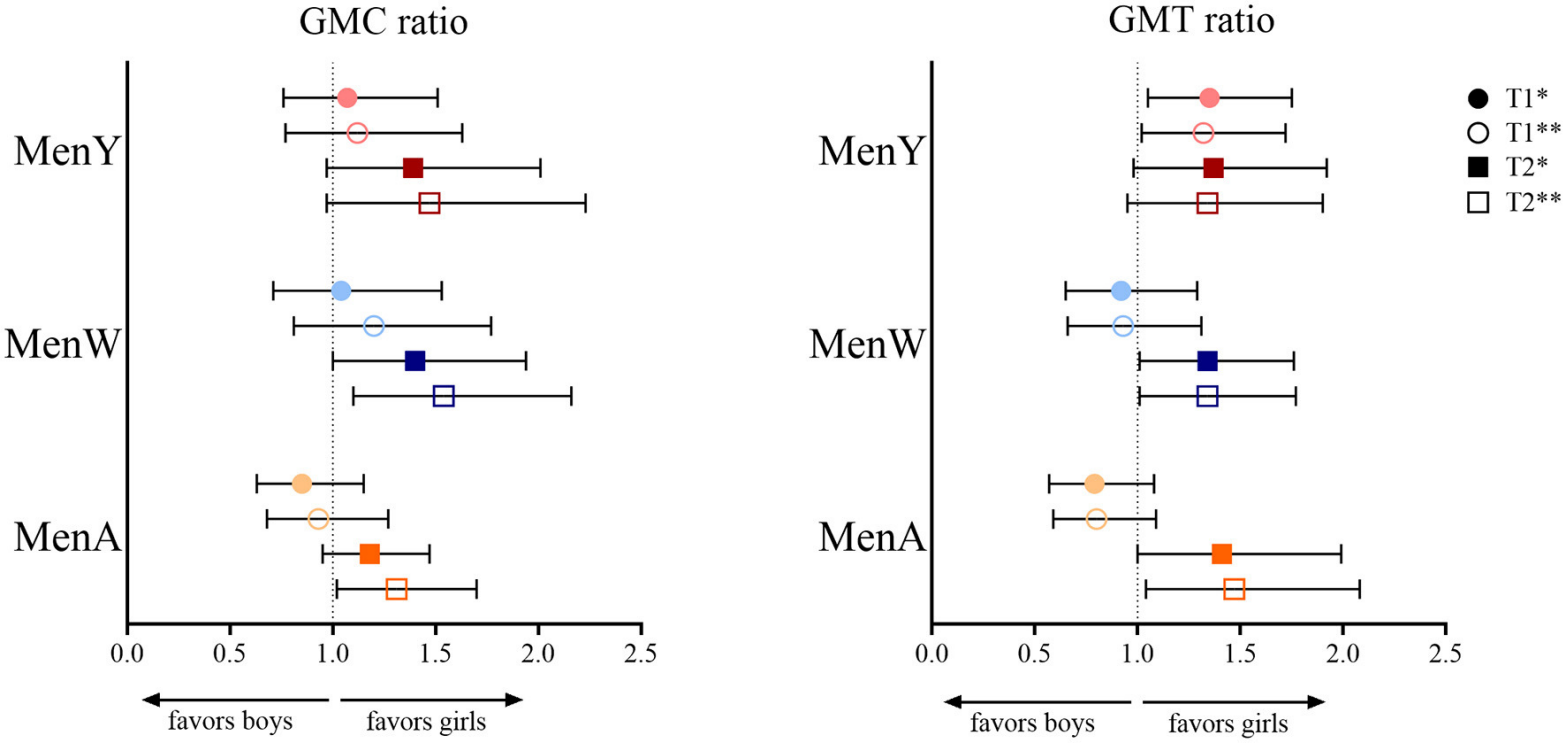
Geometric mean concentration (GMC) ratio and geometric mean titer (GMT) ratio for meningococcal serogroup A (MenA), MenW and MenY in girls vs. boys at 1 month (T1) and 1 year (T2) after a meningococcal serogroup A, C, W and Y conjugated to tetanus toxoid (MenACWY-TT) vaccine in adolescents who were primed at young age (aged between 14 months and 6 years) with a MenC-TT vaccine. *Adjusted for age group and baseline level at T0 (IgG or SBA, respectively, for GMC and GMT ratio); **adjusted for age group.

The IgG GMCs and SBA GMTs for MenA, MenW and MenY for girls and boys (across age groups) at 1 month and 1 year after booster vaccination, and the corresponding (adjusted) GMC ratios and GMT ratios are shown in Table 2A. At 1 month after the MenACWY vaccination, IgG levels and SBA titers did not differ consistently between sexes, as shown in Figure 1. Adjustment for IgG level/SBA titer at baseline slightly changed some estimates, but did not alter the observed trend.

**Table 2A:** Geometric mean IgG concentrations and geometric mean SBA titers for girls and boys and geometric mean concentration/titer ratios for girls vs. boys for meningococcal serogroups A, W and Y at 1 month and 1 year following MenACWY-TT vaccination.

	**Girls (*****n*** **=** **121)**			**Boys (*****n*** **=** **116)**			**GMC ratio (95% CI)**		**GMT ratio (95% CI)**	
	***n***	**GMC** **(95% CI)**	**GMT** **(95%CI)**	***n***	**GMC** **(95% CI)**	**GMT** **(95% CI)**	**Adjusted for** **age group**	Adjusted for age group and IgG at T0^*^	**Adjusted for** **age group**	Adjusted for age group and SBA at T0^*^
**MenA**										
T1	119	25.6 (20.4–32.2)	3482 (2,614–4,638)	113	28.5 (22.7–35.7)	4,600 (4,045–5,232)	0.93 (0.68–1.27)	0.85 (0.63–1.15)	0.80 (0.59–1.09)	0.79 (0.57–1.08)
T2	116	7.55 (6.28–9.08)	914 (711–1,176)	111	5.77 (4.78–6.96)	649 (506–832)	**1.31 (1.02–1.70)**	1.18 (0.95–1.47)	**1.47 (1.04–2.08)**	**1.41 (1.00–1.99)**
**MenW**										
T1	119	5.11 (3.80–6.88)	5,449 (3,982–7,456)	113	4.69 (3.51–6.27)	6,267 (5,377–7,305)	1.20 (0.81–1.77)	1.04 (0.71–1.53)	0.93 (0.66–1.31)	0.92 (0.65–1.29)
T2	116^a^	3.91 (3.00–5.10)	1,311 (1,026–1,676)	111	2.62 (2.10–3.27)	1,041 (892–1,214)	**1.54 (1.10–2.16)**	**1.40 (1.00–1.94)**	**1.34 (1.01–1.77)**	**1.34 (1.01–1.76)**
**MenY**										
T1	119	5.98 (4.55–7.85)	4,408 (3,510–5,534)	113	5.60 (4.27–7.33)	3,535 (3,055–4,091)	1.12 (0.77–1.63)	1.07 (0.76–1.51)	**1.32 (1.02–1.72)**	**1.35 (1.05–1.75)**
T2	116	1.98 (1.48–2.64)	1,501 (1,134–1,986)	111	1.40 (1.03–1.92)	1,183 (955–1,466)	1.47 (0.97–2.23)	1.39 (0.97–2.01)	1.34 (0.95–1.90)	1.37 (0.98–1.92)

*MenA, meningococcal serogroup A; MenW, meningococcal serogroup W135; MenY, meningococcal serogroup Y; IgG, immunoglobulin G; SBA, serum bactericidal antibody; GMC, geometric mean concentration; GMT, geometric mean titer; CI, confidence interval; T0, before vaccination; T1, 1 month after vaccination; T2, 1 year after vaccination. Significant results (p <0.05) are outlined in bold.*

* *Number of girls (F) and boys (M) excluded from the analysis due to missing IgG/SBA at T0: MenA IgG T1: 2F, 1M; MenA IgG T2: 2F, 1M; MenW IgG T1: 1F; MenW IgG T2: 1F; MenY IgG T1: 1F; MenY IgG T2: 1F; MenA SBA T1: 3F; MenA SBA T2: 3F; MenW SBA T1: 2F; MenW SBA T2: 2F; MenY SBA T1: 3F; MenY SBA T2: 3F.*

a *Number of girls included in the GMT: n = 115 (one missing SBA)*.

At 1 year after vaccination, all GMC/GMT ratio estimates were in favor of girls: ratio estimates ranged from 1.31 (1.02–1.70) for MenA IgG to 1.54 (1.10–2.16) for MenW IgG. Estimates were somewhat attenuated after adjusting for IgG/SBA at T0, e.g., to 1.18 (0.95–1.47) and 1.40 (1.00–1.94), respectively for the previously mentioned GMC ratios for MenA and MenW IgG.

### Meningococcal Serogroup C: IgG and SBA

**Figure 2:**
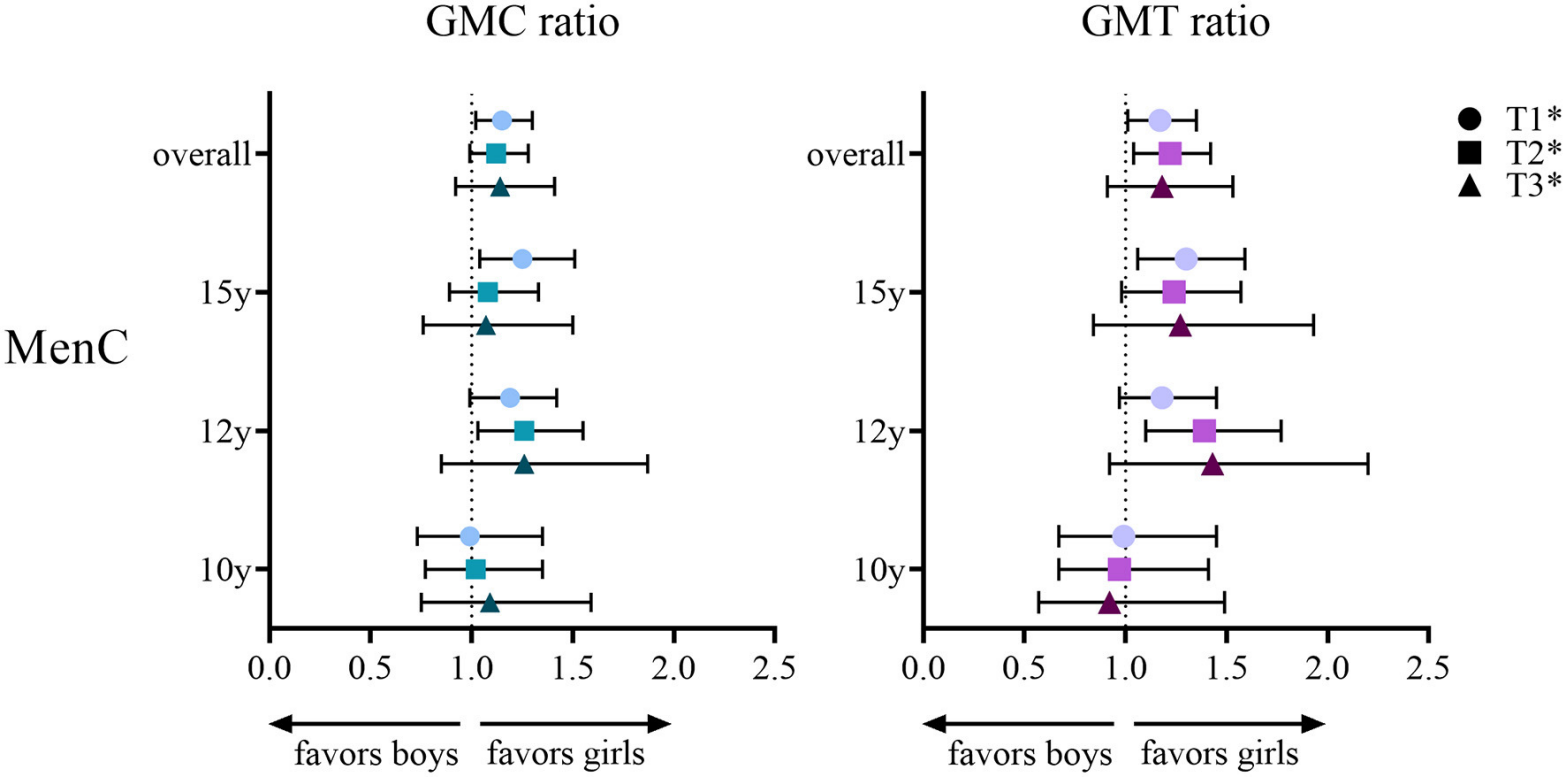
Geometric mean concentration (GMC) ratio and geometric mean titer (GMT) ratio for meningococcal serogroup C (MenC) in girls vs. boys per age group (10, 12 or 15 years) and overall at 1 month (T1), 1 year (T2) and 3 years (T3) after either a meningococcal serogroup A, C, W and Y conjugated to tetanus toxoid (MenACWY-TT) vaccine or a MenC-TT vaccine in adolescents who were primed at young age (aged between 14 months and 6 years) with a MenC-TT vaccine. *Adjusted for age group and baseline level at T0 (IgG or SBA respectively for GMC and GMT ratio).

For MenC, IgG GMCs and SBA GMTs are shown in Table 2B. Overall, both IgG and SBA were higher in girls at all postvaccination timepoints (Figure 2), e.g., at 1-month after the booster the overall IgG GMC ratio was 1.16 (1.02–1.31) and the overall SBA GMT ratio was 1.17 (1.01–1.35). When separated by age group, higher MenC IgG levels and SBA titers were observed in 12-and 15-year-old girls than in boys, whereas 10-year-old boys and girls had similar IgG levels and SBA titers.

**Table 2B:** Geometric mean IgG concentrations and geometric mean SBA titers for girls and boys and geometric mean concentration/titer ratios for girls vs. boys for meningococcal serogroup C at 1 month, 1 year and 3 years following MenC-TT/MenACWY-TT booster vaccination.

**MenC**	**Girls (*****n*** **=** **121)**				**Boys (*****n*** **=** **116)**				**GMC ratio (95% CI)**		**GMT ratio (95% CI)**	
	***n* (IgG)**	***n* (SBA)**	**GMC** **(95%CI)**	**GMT** **(95%CI)**	***n* (IgG)**	***n* (SBA)**	**GMC** **(95%CI)**	**GMT** **(95%CI)**	**adjusted for study group**	adjusted for study group and IgG at T0^*^	**adjusted for** **study group**	adjusted for study group and SBA at T0^*^
**T1**
10 y	97	97	124 (98.6–156)	29,012 (21,723–38,748)	68	68	124 (103–150)	29,079 (23,331–36,244)	1.00 (0.74–1.37)	0.99 (0.73–1.35)	1.00 (0.68–1.47)	0.99 (0.67–1.45)
12 y	113	112	191 (167–219)	41,167 (35,033–48,374)	133	133	160 (142–181)	34,586 (30,143–39,683)	1.19 (0.99–1.42)	1.19 (0.99–1.42)	1.18 (0.96–1.45)	1.18 (0.97–1.45)
15 y	122	121	184 (162–209)	43,726 (38,179–50,078)	121	121	147 (128–169)	34,428 (29,264–40,503)	**1.24 (1.03–1.49)**	**1.25 (1.04–1.51)**	**1.27 (1.04–1.56)**	**1.30 (1.06–1.59)**
Overall	332	330	166 (151–183)	37,973 (33,914–42,519)	322	322	147 (136–160)	33,285 (30,276–36,592)	**1.16 (1.02–1.31)**	**1.15 (1.02–1.30)**	**1.17 (1.01–1.35)**	**1.17 (1.01–1.35)**
**T2**
10 y	93	93	9.73 (7.91–12.0)	1,523 (1,159–2,002)	66	66	9.66 (7.77–12.0)	1,581 (1,217–2,054)	1.02 (0.76–1.36)	1.02 (0.77–1.35)	0.97 (0.67–1.42)	0.97 (0.67–1.41)
12 y	111	110	17.5 (14.7–20.7)	3,355 (2,755–4,085)	132	132	13.7 (11.5–16.2)	2,383 (1,994–2,848)	**1.26 (1.01–1.57)**	**1.26 (1.03–1.55)**	**1.38 (1.08–1.77)**	**1.39 (1.10–1.77)**
15 y	121	121	25.6 (22.0–29.9)	4,780 (4,007–5,701)	117	117	22.3 (19.0–26.1)	3,871 (3,221–4,651)	1.16 (0.94–1.42)	1.08 (0.89–1.33)	1.24 (0.98–1.58)	1.24 (0.98–1.57)
Overall	325	324	17.0 (15.3–19.0)	3,052 (2,677–3,480)	315	315	15.2 (13.7–17.0)	2,619 (2,323–2,952)	**1.16 (1.01–1.32)**	1.12 (0.99–1.28)	**1.22 (1.04–1.43)**	**1.22 (1.04–1.42)**
**T3**
10 y	40	40	6.27 (4.82–8.16)	578 (404–827)	26	26	6.62 (5.22–8.40)	686 (533–884)	0.95 (0.66–1.36)	1.09 (0.75–1.59)	0.84 (0.53–1.34)	0.92 (0.57–1.49)
12 y	37	37	15.3 (12.1–19.5)	2,335 (1,670–3,264)	38	38	12.4 (8.54–17.9)	1,707 (1,202–2,422)	1.24 (0.81–1.89)	1.26 (0.85–1.87)	1.37 (0.86–2.17)	1.43 (0.92–2.20)
15 y	33	33	20.7 (15.9–27.0)	4,096 (2,994–5,603)	25	25	17.5 (13.6–22.5)	3,191 (2,373–4,293)	1.19 (0.83–1.69)	1.07 (0.76–1.50)	1.28 (0.85–1.95)	1.27 (0.84–1.93)
Overall	110	110	12.1 (10.2–14.4)	1,663 (1,300–2,129)	89	89	11.4 (9.32–13.8)	1,559 (1,253–1,940)	1.12 (0.90–1.41)	1.14 (0.92–1.41)	1.15 (0.88–1.50)	1.18 (0.91–1.53)

*MenC, meningococcal serogroup C; IgG, immunoglobulin G; SBA, serum bactericidal antibody; GMC, geometric mean concentration; GMT, geometric mean titer; CI, confidence interval; T0, before vaccination; T1, 1 month after vaccination; T2, 1 year after vaccination; T3, 3 years after vaccination. Significant results (p <0.05) are outlined in bold.*

* *Number of girls (F) and boys (M) excluded from the analysis due to missing IgG/SBA at T0: IgG T1 10y: 1F; IgG T1 overall: 1F; IgG T2 10y: 1F IgG T2 overall: 1F; SBA T1 10y: 1F; SBA T1 15y: 1F; SBA T1 overall: 2F; SBA T2 10y: 1F; SBA T2 15y: 1F; SBA T2 overall: 2F*.

### Meningococcal Serogroup A, C, W and Y: Proportions Protected

The vast majority of participants (96–100%), both girls and boys, were protected against all serogroups 1 month and 1 year after vaccination. There were no significant differences in the proportions protected (SBA ≥ 8) between girls and boys at any timepoint or for any serogroup (Table 3). Three years after vaccination, all girls and boys (*n* = 110 and *n* = 89, respectively) were still protected against MenC (18).

**Table 3:** Proportions protected according to SBA titer for girls and boys for all serogroups and timepoints.

**Serogroup**	**Timepoint**	**Girls**			**Boys**			*p*-value^*^
		***N* protected (SBA ≥8)**	***N* total**	**%**	***N* protected (SBA ≥8)**	***N* total**	**%**	
A	1 mo	115	119	96.6	113	113	100	0.122
	1 yr	112	116	96.6	107	111	96.4	1.000
W	1 mo	115	119	96.6	113	113	100	0.122
	1 yr	112	115	97.4	111	111	100	0.247
Y	1 mo	118	119	99.2	113	113	100	1.000
	1 yr	112	116	96.6	110	111	99.1	0.370
C	1 mo	329	330	99.7	322	322	100	1.000
	1 yr	322	324	99.4	315	315	100	0.499
	3 yr^a^	110	110	100	89	89	100	NA

*mo, month; yr, year; NA, not applicable.*

* *p-values (two-sided) of the difference in proportion protected between girls and boys were determined with Fisher's exact test.*

a *Determined in a subgroup of participants who participated in a follow-up study*.

### Serum and Salivary Meningococcal IgA

Results for serum IgA and salivary IgA are shown in Supplementary Tables 2A,B (with baseline characteristics in Supplementary Tables 1A,B). The observed trend of the IgA results was similar to IgG and SBA, either showing no clear difference or somewhat higher levels in girls. However, although the trend was similar, the difference only reached significance for MenY serum IgA at T2 when adjusted for age group only (*p* = 0.037) or for age group and baseline levels (*p* = 0.019). For MenC, a significant difference toward girls was observed for serum IgA at 1 year after vaccination in 12-year-olds, when adjusted for age and baseline level. A significant difference was found for MenC salivary IgA at 1 month after vaccination in 12-year-olds as well as for the overall group.

### Serum Tetanus IgG

Results for TT-specific serum IgG were only available for the MenC-booster group for baseline, 1 month and 1 year after vaccination (Supplementary Table 3). We found a significant difference for 10-year-olds at T2 with a higher level in boys [GMC ratio girls vs. boys: 0.72 (0.54–0.96), *p* = 0.024], but we found no significant difference in other age groups nor at other timepoints.

## Discussion

In this study, we evaluated sex-related differences in the immune response to a meningococcal conjugate vaccine in adolescents. We found slightly higher antibody levels in girls than in boys at the age of 12 or 15 years, respectively, and at more than a month after vaccination. Our results suggest some sex-based disparity in the meningococcal vaccine-induced immune response during adolescence. Since this is a period characterized by a developing and changing hormonal system while simultaneously being prone for carriage of meningococci, a sufficient vaccine response is important.

To our knowledge, we are the first to report meningococcal vaccine-induced sex-specific immune responses in adolescents. A meta-analysis by Voysey et al. (9) found consistently higher immune responses in girls than boys-all aged younger than 3 years-to a [diphtheria cross-reacting material (CRM197) conjugated] meningococcal ACWY vaccine for serogroup A, W and Y, but not for serogroup C with most geometric mean MenC ratios close to 1. This is in contrast to our results that showed favorable results in girls for all serogroups including serogroup C, albeit not for each timepoint. In line with our findings, a study that investigated the vaccine response to other capsular conjugate vaccines like the pneumococcal vaccine and Haemophilus influenza type B (Hib) vaccine reported no differences or higher antibody levels in females, although they included infants and young children (27). Similar, a trend of comparable or higher tetanus antibody levels in boys was also observed in that study.

It was previously proposed that the carrier protein in conjugate vaccines might have a sex-differential effect (9, 28). In the current study, all participants received a meningococcal vaccine conjugated to tetanus toxoid and we could not make a comparison between different carrier proteins. Yet, with regard to the carrier protein itself, we only identified a significant difference in 10-year-olds with higher tetanus antibody levels in boys rather than girls. This finding does not prove nor exclude a sex-differential effect toward females as promoted by the carrier protein.

Since age is inextricably linked with sex hormones-which induce variation of the immune profile during life-the influence of age should always be considered in studies comparing responses according to sex. Generally, estrogens have a variable (mostly activating) effect on the immune function, while progesterone is considered as a modulator or suppressive hormone and testosterone mainly acts as immunosuppressor (2, 29). In adolescence, the actions of steroid hormones result in extensive changes to an individual's body (30), including the immune system. Therefore, our results cannot be translated directly to younger children or elderly, in whom sex-differences are hypothesized to be minimized due to the life-course related changing hormonal status. For instance, the effect of sex could be limited in postmenopausal women due to relatively high levels of progesterone compared to earlier in life (and comparable progesterone levels to males at elder age), though genetic differences continue to exist. This is also highlighted by the fact that we found sex-differences in 12- and 15-year-olds but not in 10-year-olds. At the age of 10, most children are in a phase prior to, or at the start of the pubertal rise of reproductive hormones that is called the gonadarche (31, 32). Before this phase, the effect of gonadal steroids on the vaccine response is expected to be limited. The implications of sex-differential effects for vaccination policy are therefore dependent on many factors and sex should always be considered in relation to age.

Not only vaccination or disease, but also asymptomatic carriage can induce the production of antibodies (33, 34). We cannot exclude that carriage of the bacterium might have influenced our results, since serogroup C, W and Y are still prevalent in the Netherlands (13). To what extent carriage might have affected our results remains uncertain, but evidence for sex-differential meningococcal carriage rates is limited. A large carriage study in the UK that investigated predisposing factors for meningococcal carriage in teenagers did not find an association between carriage and sex (35), similar to results from a study in adolescents in Australia (36). In university students in the United States, meningococcal carriage was in fact associated with being male (37). In this study, we did not find any significant sex-related differences in IgA levels. IgA is the dominant Ig type in mucosal tissues and thus important in the first line of defense at the location of carriage, e.g., the nasopharynx and its mucosal surfaces (38, 39). Moreover, the sex-related differences in IgG levels we found were present after vaccination, but not before vaccination and carriage levels are known to increase after the age of 15 years (14). Therefore, it appears unlikely that our results were confounded by naturally-acquired immunity.

In spite of a difference in geometric mean antibody levels, protection levels did not differ significantly up to 3 years postvaccination. Longer follow-up studies are necessary to investigate the implications for adolescents when antibodies wane. Although there seems to be a tendency of faster waning for MenAWY in males (with sex differences increasing over time), we did not observe this pattern for MenC. We have some data available 5 years after the MenACWY vaccination for a subgroup of participants, but these results were inconclusive due to small sample sizes and proportions protected were still very high among adolescents as was previously published (40). We encourage future clinical trials, carriage studies but also serosurveillance studies-that often cover longer periods after vaccination due to the nature of the study-to report data stratified by sex. Thereby, the knowledge on sex differences in vaccine-induced immune responses could be expanded, not only for meningococci but also for other vaccine-preventable pathogens. Meningococcal vaccination policy might not change when long-term data would become available, which is supported by our finding that differences between sexes are limited 3 years postvaccination and protection levels at that timepoint were very high for both sexes. However, it might be relevant for other vaccine-preventable diseases if vaccine-induced immunity wanes fast in one sex but not the other.

One of the strengths of this study is the clinical trial setting of the studies with a fairly equal number of included boys and girls enabled *post-hoc* analysis without risk of selection bias. We investigated both IgG concentrations and functional antibody titers, which enabled analysis of the proportions protected next to geometric means of antibody levels. However, despite our trial has a follow-up time of 3 years, we found very high levels of protected participants at this latest timepoint. This hampered the exploration of clinical relevance of the biological differences that we found. Future modeling studies could estimate potential differences in duration of protection and serosurveillance studies should also consider presenting data by sex to explore sex-based differences in antibody levels across the population. One of the limitations of the study is the lack of information about every individual's pubertal maturation status at time of the study. Since the onset of puberty differs per individual, we could not analyse the results per puberty stage (pre-puberty vs. puberty) next to the age-specific analyses that we did. Furthermore, we could not analyse the MenAWY results per age group due to the limited number of participants in the MenACWY booster group. Nevertheless, we did have a large sample size in the MenC booster group which enabled us to examine MenC IgG, IgA and TT results per age group.

To conclude, our data showed that the vaccine responses following an adolescent MenC or MenACWY vaccination were slightly higher in 12- and 15-year-old girls than in boys. However, the percentage with protective titers was very high for both boys and girls. More research is needed to establish whether these findings are of clinical relevance on the long-term when antibodies wane and protection levels decrease.

## Data Availability Statement

The original contributions presented in the study are included in the article/Supplementary Materials, further inquiries can be directed to the corresponding author/s.

## Ethics Statement

The studies involving human participants were reviewed and approved by VCMO. Written informed consent to participate in this study was provided by the participants' legal guardian/next of kin.

## Author Contributions

MO, AB, MR, GB, and MK were involved in the conception, planning, and study design. SS and MR performed the laboratory analyses. MO and AB performed statistical analyses with input from MK. MO, GB, and MK interpreted data and wrote the manuscript. All authors critically reviewed the manuscript, approved the final version, have contributed significantly to the work, and agreed to submit for publication.

## Funding

This work was supported by the Dutch Ministry of Health, Welfare and Sport in the framework of RIVM Strategic Programme (SPR), that focusses on themes to innovate and develop knowledge, and prepare RIVM to respond to future issues in health and sustainability with expertise and innovative projects.

## Conflict of Interest

The authors declare that the research was conducted in the absence of any commercial or financial relationships that could be construed as a potential conflict of interest.

## Publisher's Note

All claims expressed in this article are solely those of the authors and do not necessarily represent those of their affiliated organizations, or those of the publisher, the editors and the reviewers. Any product that may be evaluated in this article, or claim that may be made by its manufacturer, is not guaranteed or endorsed by the publisher.

## References

[1] Taneja V. Sex hormones determine immune response. Front Immunol. (2018) 9:1931. 10.3389/fimmu.2018.01931 30210492 PMC6119719

[2] Klein SL Flanagan KL. Sex differences in immune responses. Nat Rev Immunol. (2016) 16:626–38. 10.1038/nri.2016.90 27546235

[3] Rosenstein NE Perkins BA Stephens DS Popovic T Hughes JM. Meningococcal Disease. N Eng J Med. (2001) 344:1378–88. 10.1056/NEJM200105033441807 11333996

[4] Green MS Schwartz N Peer V. A meta-analytic evaluation of sex differences in meningococcal disease incidence rates in 10 countries. Epidemiol Infect. (2020) 148:e246. 10.1017/S0950268820002356 33004098 PMC7592104

[5] Loenenbach AD van der Ende A de Melker HE Sanders EAM Knol MJ. The clinical picture and severity of invasive meningococcal disease serogroup w compared with other serogroups in the Netherlands, 2015-2018. Clin Infect Dis. (2020) 70:2036–44. 10.1093/cid/ciz578 31556938 PMC7201410

[6] Bloch D Murray K Peterson E Ngai S Rubinstein I Halse TA. Sex difference in meningococcal disease mortality, New York City, 2008–2016. Clin Infect Dis. (2018) 67:760–9. 10.1093/cid/ciy183 29509877

[7] Geller SE Koch AR Roesch P Filut A Hallgren E Carnes M. The more things change, the more they stay the same: a study to evaluate compliance with inclusion and assessment of women and minorities in randomized controlled trials. Acad Med. (2018) 93:630–5. 10.1097/ACM.0000000000002027 29053489 PMC5908758

[8] Franconi F Campesi I Colombo D Antonini P. Sex-gender variable: methodological recommendations for increasing scientific value of clinical studies. Cells. (2019) 8:476. 10.3390/cells8050476 31109006 PMC6562815

[9] Voysey M Barker CIS Snape MD Kelly DF Trück J Pollard AJ. Sex-dependent immune responses to infant vaccination: an individual participant data meta-analysis of antibody and memory B cells. Vaccine. (2016) 34:1657–64. 10.1016/j.vaccine.2016.02.036 26920472

[10] Klein SL Pekosz A. Sex-based biology and the rational design of influenza vaccination strategies. J Infect Dis. (2014) 209:S114–9. 10.1093/infdis/jiu066 24966191 PMC4157517

[11] de Voer RM Mollema L Schepp RM de Greeff SC van Gageldonk PG de Melker HE. Immunity against Neisseria meningitidis serogroup C in the Dutch population before and after introduction of the meningococcal c conjugate vaccine. PLoS ONE. (2010) 5:e12144. 10.1371/journal.pone.0012144 20730091 PMC2921331

[12] Knol MJ Ruijs WL Antonise-Kamp L de Melker HE van der Ende A. Implementation of MenACWY vaccination because of ongoing increase in serogroup W invasive meningococcal disease, the Netherlands, 2018. Eurosurveillance. (2018) 23:18–00158. 10.2807/1560-7917.ES.2018.23.16.18-00158 29692317 PMC5915972

[13] Ohm M Hahné SJM van der Ende A Sanders EAM Berbers GAM Ruijs WLM. Vaccine impact and effectiveness of meningococcal serogroup ACWY conjugate vaccine implementation in the Netherlands: a nationwide surveillance study. Clin Infect Dis. (2021). 10.1093/cid/ciab791 [Epub ahead of print]. PMC9258937 34525199

[14] Christensen H May M Bowen L Hickman M Trotter CL. Meningococcal carriage by age: a systematic review and meta-analysis. Lancet Infect Dis. (2010) 10:853–61. 10.1016/S1473-3099(10)70251-6 21075057

[15] van Ravenhorst MB van der Klis FRM van Rooijen DM Knol MJ Stoof SP Sanders EAM. Meningococcal serogroup C immunogenicity, antibody persistence and memory B-cells induced by the monovalent meningococcal serogroup C versus quadrivalent meningococcal serogroup ACWY conjugate booster vaccine: a randomized controlled trial. Vaccine. (2017) 35:4745–52. 10.1016/j.vaccine.2017.06.053 28668575

[16] van Ravenhorst MB van der Klis FRM van Rooijen DM Sanders EAM Berbers GAM. Adolescent meningococcal serogroup A, W and Y immune responses following immunization with quadrivalent meningococcal A, C, W and Y conjugate vaccine: optimal age for vaccination. Vaccine. (2017) 35:4753–60. 10.1016/j.vaccine.2017.06.007 28647167

[17] Stoof SP van der Klis FR van Rooijen DM Knol MJ Sanders EA Berbers GA. Timing of an adolescent booster after single primary meningococcal serogroup C conjugate immunization at young age; an intervention study among Dutch teenagers. PLoS ONE. (2014) 9:e100651. 10.1371/journal.pone.0100651 24963638 PMC4070982

[18] van Ravenhorst MB Marinovic AB van der Klis FR van Rooijen DM van Maurik M Stoof SP. Long-term persistence of protective antibodies in Dutch adolescents following a meningococcal serogroup C tetanus booster vaccination. Vaccine. (2016) 34:6309–15. 10.1016/j.vaccine.2016.10.049 27817957

[19] de Voer RM Schepp RM Versteegh FG van der Klis FR Berbers GA. Simultaneous detection of haemophilus influenzae type b polysaccharide-specific antibodies and neisseria meningitidis serogroup A, C, Y, and W-135 polysaccharide-specific antibodies in a fluorescent-bead-based multiplex immunoassay. Clin Vaccine Immunol. (2009) 16:433–6. 10.1128/CVI.00364-08 19129470 PMC2650869

[20] de Voer RM van der Klis FR Engels CW Rijkers GT Sanders EA Berbers GA. Development of a fluorescent-bead-based multiplex immunoassay to determine immunoglobulin G subclass responses to Neisseria meningitidis serogroup A and C polysaccharides. Clin Vaccine Immunol. (2008) 15:1188–93. 10.1128/CVI.00478-07 18550729 PMC2519294

[21] Lal G Balmer P Joseph H Dawson M Borrow R. Development and evaluation of a tetraplex flow cytometric assay for quantitation of serum antibodies to Neisseria meningitidis serogroups A, C, Y, and W-135. Clin Diagn Lab Immunol. (2004) 11:272–9. 10.1128/CDLI.11.2.272-279.2004 15013975 PMC371201

[22] van Gageldonk PG van Schaijk FG van der Klis FR Berbers GA. Development and validation of a multiplex immunoassay for the simultaneous determination of serum antibodies to Bordetella pertussis, diphtheria and tetanus. J Immunol Methods. (2008) 335:79–89. 10.1016/j.jim.2008.02.018 18407287

[23] Maslanka SE Gheesling LL Libutti DE Donaldson KB Harakeh HS Dykes JK. Standardization and a multilaboratory comparison of Neisseria meningitidis serogroup A and C serum bactericidal assays. the multilaboratory study group. Clin Diagn Lab Immunol. (1997) 4:156–67. 10.1128/cdli.4.2.156-167.1997 9067649 PMC170495

[24] Borrow R Andrews N Goldblatt D Miller E. Serological basis for use of meningococcal serogroup C conjugate vaccines in the United Kingdom: reevaluation of correlates of protection. Infect Immun. (2001) 69:1568–73. 10.1128/IAI.69.3.1568-1573.2001 11179328 PMC98057

[25] Borrow R Balmer P Miller E. Meningococcal surrogates of protection–serum bactericidal antibody activity. Vaccine. (2005) 23:2222–7. 10.1016/j.vaccine.2005.01.051 15755600

[26] Andrews N Borrow R Miller E. Validation of serological correlate of protection for meningococcal C conjugate vaccine by using efficacy estimates from postlicensure surveillance in England. Clin Diagn Lab Immunol. (2003) 10:780–6. 10.1128/CDLI.10.5.780-786.2003 12965904 PMC193909

[27] Boef AGC van der Klis FRM Berbers GAM Buisman AM Sanders EAM Kemmeren JM. Differences by sex in IgG levels following infant and childhood vaccinations: an individual participant data meta-analysis of vaccination studies. Vaccine. (2018) 36:400–7. 10.1016/j.vaccine.2017.11.070 29223483

[28] Knuf M Kowalzik F Kieninger D. Comparative effects of carrier proteins on vaccine-induced immune response. Vaccine. (2011) 29:4881–90. 10.1016/j.vaccine.2011.04.053 21549783

[29] Fischinger S Boudreau CM Butler AL Streeck H Alter G. Sex differences in vaccine-induced humoral immunity. Semin Immunopathol. (2019) 41:239–49. 10.1007/s00281-018-0726-5 30547182 PMC6373179

[30] Hansen AB Wøjdemann D Renault CH Pedersen AT Main KM Raket LL. Diagnosis of endocrine disease: Sex steroid action in adolescence: too much, too little; too early, too late. Eur J Endocrinol. (2021) 184:R17–r28. 10.1530/EJE-20-0545 33112274

[31] Viner RM Allen NB Patton GC. Puberty, developmental processes, and health interventions. In: Bundy DAP, Silva ND, Horton S, Jamison DT, Patton GC, editors. Child and Adolescent Health and Development. Washington, DC: The International Bank for Reconstruction and Development / The World Bank 2017 (2017). 10.1596/978-1-4648-0423-6_ch9 30212144

[32] Levesque RJR. Gonadarche. In: Levesque RJR, editor. Encyclopedia of Adolescence. New York, NY: Springer New York (2011). p. 1196. 10.1007/978-1-4419-1695-2_551

[33] Goldschneider I Gotschlich EC Artenstein MS. Human immunity to the meningococcus. II development of natural immunity. J Exp Med. (1969) 129:1327–48. 10.1084/jem.129.6.1327 4977281 PMC2138665

[34] Jordens JZ Williams JN Jones GR Christodoulides M Heckels JE. Development of immunity to serogroup B meningococci during carriage of Neisseria meningitidis in a cohort of university students. Infect Immun. (2004) 72:6503–10. 10.1128/IAI.72.11.6503-6510.2004 15501781 PMC523012

[35] MacLennan J Kafatos G Neal K Andrews N Cameron JC Roberts R. Social behavior and meningococcal carriage in British teenagers. Emerg Infect Dis. (2006) 12:950–7. 10.3201/eid1206.051297 16707051 PMC3373034

[36] Marshall HS McMillan M Koehler AP Lawrence AJ Sullivan TR Maclennan JM. Meningococcal B vaccine and meningococcal carriage in adolescents in Australia. N Eng J Med. (2020) 382:318–27. 10.1056/NEJMoa1900236 31971677

[37] Breakwell L Whaley M Khan UI Bandy U Alexander-Scott N Dupont L. Meningococcal carriage among a university student population - United States, 2015. Vaccine. (2018) 36:29–35. 10.1016/j.vaccine.2017.11.040 29183735 PMC5737556

[38] Pabst O Cerovic V Hornef M. Secretory IgA in the Coordination of establishment and maintenance of the microbiota. Trends Immunol. (2016) 37:287–96. 10.1016/j.it.2016.03.002 27066758

[39] Jarvis GA Griffiss JM. Human IgA1 initiates complement-mediated killing of Neisseria meningitidis. J Immunol. (1989) 143:1703–9. 2474610

[40] Ohm M van Rooijen DM Bonačić Marinović AA van Ravenhorst MB van der Heiden M Buisman AM. Different long-term duration of seroprotection against neisseria meningitidis in adolescents and middle-aged adults after a single meningococcal acwy conjugate vaccination in the Netherlands. Vaccines. (2020) 8:624. 10.3390/vaccines8040624 33113834 PMC7712102

