# Supplementary material for: Sex-Related Differences in the Immune Response to Meningococcal Vaccinations During Adolescence
Source: Front Public Health. 2022 May 6;10:871670. doi: 10.3389/fpubh.2022.871670 (PMC9120633; doi:10.3389/fpubh.2022.871670)
Supplement: Supplementary file 1 [file Table_1.DOCX]

**Supplementary Table 1A Characteristics of the study population for meningococcal serogroups A, W and Y (IgA analyses).**

| Characteristic | Girls (n=121) | Boys (n=116) |
| --- | --- | --- |
| Baseline serum IgA in µg/mL,  median (IQR) |  |  |
| MenA | 0.075 (0.039-0.160) *(n=114)* | 0.064 (0.032-0.115) *(n=113)* |
| MenW | 0.017 (0.007-0.046) *(n=114)* | 0.019 (0.001-0.037) *(n=113)* |
| MenY | 0.044 (0.007-0.097) *(n=114)* | 0.036 (0.014-0.068) *(n=111)* |
| Baseline salivary IgA in ng/mL,  median (IQR) |  |  |
| MenA | 42 (26-65) *(n=118)* | 38 (26-61) *(n=112)* |
| MenW | 13 (11-17) *(n=120)* | 13 (10-16) *(n=115)* |
| MenY | 28 (20-38) *(n=120)* | 25 (18-33) *(n=115)* |

Abbreviations: MenA, meningococcal serogroup A; MenW, meningococcal serogroup W135; MenY, meningococcal serogroup Y; IgA immunoglobulin A; IQR, interquartile range.

**Supplementary Table 1B Characteristics of the study population for meningococcal serogroup C (IgA analyses).**

| Characteristic | | Girls (n=342) | Boys (n=327) |
| --- | --- | --- | --- |
| Baseline IgA, median (IQR) | |  |  |
| Serum (µg/mL) | |  |  |
| Overall | | 0.025 (0.010-0.064) *(n=328)* | 0.022 (0.010-0.055) *(n=322)* |
| 10y | 14m | 0.013 (0.006-0.036) *(n=95)* | 0.012 (0.003-0.032) *(n=69)* |
| 12y | 3y | 0.016 (0.009-0.043) *(n=41)* | 0.020 (0.007-0.104) *(n=47)* |
| 12y | 14m | 0.027 (0.013-0.041) *(n=71)* | 0.027 (0.012-0.054) *(n=87)* |
| 15y | 6y | 0.054 (0.014-0.147) *(n=40)* | 0.020 (0.010-0.093) *(n=43)* |
| 15y | 3y | 0.044 (0.020-0.091) *(n=81)* | 0.032 (0.017-0.064) *(n=76)* |
| Saliva (ng/mL) | |  |  |
| Overall | | 7.7 (5.0-12.3) *(n=336)* | 7.6 (5.0-12.1) *(n=324)* |
| 10y | 14m | 6.9 (4.0-9.2) *(n=96)* | 5.8 (3.6-9.3) *(n=71)* |
| 12y | 3y | 6.0 (3.0-12.3) *(n=44)* | 5.8 (3.5-12.6) *(n=47)* |
| 12y | 14m | 7.7 (5.4-12.0) *(n=71)* | 7.6 (5.4-9.6) *(n=85)* |
| 15y | 6y | 11.0 (5.8-16.2) *(n=41)* | 9.0 (4.2-18.4) *(n=45)* |
| 15y | 3y | 9.0 (6.3-15.0) *(n=84)* | 10.2 (6.9-17.9) *(n=76)* |

Abbreviations: MenC, meningococcal serogroup C; IgA immunoglobulin A; SBA, serum bactericidal antibody; IQR, interquartile range.

**Supplementary Table 2A Geometric mean serum and salivary IgA concentrations for girls and boys and geometric mean concentration ratios for girls versus boys for meningococcal serogroups A, W and Y at 1 month and 1 year following MenACWY-TT vaccination.**

|  | | Girls (n=121) | |  | Boys (n=116) | |  | GMC ratio (95% CI) | |
| --- | --- | --- | --- | --- | --- | --- | --- | --- | --- |
|  |  | n | GMC  (95% CI) |  | n | GMC  (95% CI) |  | adjusted for  age group | adjusted for age group & IgG at T0* |
| **MenA** |  |  |  |  |  |  |  |  |  |
| serum (µg/mL) | T1 | 119 | 1.40 (1.11-1.76) |  | 113 | 1.34 (1.11-1.62) |  | 1.12 (0.84-1.48) | 1.10 (0.83-1.47) |
|  | T2 | 113 | 0.32 (0.24-0.42) |  | 106 | 0.32 (0.25-0.41) |  | 1.04 (0.73-1.47) | 0.96 (0.67-1.37) |
| saliva (ng/mL) | T1 | 115 | 93.4 (80.6-108.2) |  | 112 | 85.5 (72.9-100.2) |  | 1.10 (0.89-1.37) | 1.08 (0.87-1.33) |
|  | T2 | 110 | 34.9 (29.8-41.0) |  | 103 | 31.6 (26.6-37.7) |  | 1.16 (0.92-1.45) | 1.08 (0.87-1.33) |
| **MenW** |  |  |  |  |  |  |  |  |  |
| serum (µg/mL) | T1 | 119 | 0.81 (0.62-1.06) |  | 112 | 0.87 (0.69-1.09) |  | 1.01 (0.73-1.41) | 1.03 (0.74-1.43) |
|  | T2 | 113 | 0.47 (0.38-0.59) |  | 106 | 0.39 (0.32-0.47) |  | 1.28 (0.96-1.69) | 1.27 (0.96-1.68) |
| saliva (ng/mL) | T1 | 116 | 27.1 (22.8-32.3) |  | 115 | 27.5 (22.6-33.4) |  | 1.01 (0.78-1.30) | 0.99 (0.76-1.28) |
|  | T2 | 112 | 10.1 (8.4-12.2) |  | 106 | 11.8 (9.7-14.3) |  | 0.95 (0.74-1.21) | 0.89 (0.71-1.13) |
| **MenY** |  |  |  |  |  |  |  |  |  |
| serum (µg/mL) | T1 | 120 | 1.78 (1.38-2.30) |  | 112 | 1.85 (1.43-2.38) |  | 1.05 (0.75-1.47) | 1.11 (0.80-1.54) |
|  | T2 | 111 | 0.95 (0.74-1.21) |  | 106 | 0.69 (0.54-0.90) |  | **1.43 (1.02-2.00)** | **1.48 (1.07-2.06)** |
| saliva (ng/mL) | T1 | 117 | 52.9 (46.2-60.7) |  | 115 | 47.2 (40.0-55.6) |  | 1.16 (0.94-1.42) | 1.11 (0.90-1.36) |
|  | T2 | 112 | 25.9 (22.7-29.5) |  | 106 | 24.6 (21.6-28.1) |  | 1.10 (0.93-1.31) | 1.03 (0.88-1.20) |

Abbreviations: MenA, meningococcal serogroup A; MenW, meningococcal serogroup W135; MenY, meningococcal serogroup Y; IgA immunoglobulin A; GMC, geometric mean concentration; CI, confidence interval; T0, before vaccination; T1, 1 month after vaccination; T2, 1 year after vaccination.

*Number of girls (F) and boys (M) excluded from the analysis due to missing IgA at T0: MenA serum T1: 6F, 2M; MenA serum T2: 5F, 2M; MenW serum T1: 7F, 2M; MenW serum T2: 5F, 2M; MenY serum T1: 7F, 4M; MenY serum T2: 4F, 3M; MenA saliva T1: 1M; MenW saliva T1: 1M; MenY saliva T1: 1M.

**Supplementary Table 2B. Geometric mean serum and salivary IgA concentrations and geometric mean concentration ratios for girls versus boys for meningococcal serogroup C at 1 month and 1 year following MenC-TT/MenACWY-TT booster vaccination.**

|  | Girls | |  | Boys | |  | GMC ratio (95% CI) | |
| --- | --- | --- | --- | --- | --- | --- | --- | --- |
|  | n | GMC (95% CI) |  | n | GMC (95% CI) |  | adjusted for study group | adjusted for study group and IgA at T0 |
| Serum IgA (µg/mL) |  |  |  |  |  |  |  |  |
| T1 |  |  |  |  |  |  |  |  |
| 10y | 97 | 6.73 (5.01-9.03) |  | 68 | 7.48 (5.69-9.85) |  | 0.90 (0.60-1.36) | 0.92 (0.65-1.31) |
| 12y | 112 | 14.6 (12.4-17.3) |  | 133 | 12.5 (10.4-14.9) |  | 1.18 (0.92-1.50) | 1.16 (0.92-1.47) |
| 15y | 122 | 17.2 (14.6-20.2) |  | 121 | 16.3 (13.5-19.8) |  | 1.05 (0.82-1.34) | 0.99 (0.77-1.26) |
| Overall | 331 | 12.4 (10.9-14.0) |  | 322 | 12.4 (11.0-14.0) |  | 1.05 (0.89-1.24) | 1.03 (0.89-1.21) |
| T2 |  |  |  |  |  |  |  |  |
| 10y | 90 | 0.69 (0.53-0.90) |  | 65 | 0.65 (0.49-0.86) |  | 1.06 (0.73-1.55) | 1.03 (0.73-1.46) |
| 12y | 110 | 1.89 (1.55-2.30) |  | 129 | 1.43 (1.17-1.75) |  | 1.32 (0.99-1.74) | **1.30 (1.00-1.69)** |
| 15y | 121 | 3.13 (2.57-3.80) |  | 116 | 2.70 (2.21-3.32) |  | 1.16 (0.88-1.53) | 1.07 (0.82-1.39) |
| Overall | 321 | 1.72 (1.50-1.98) |  | 310 | 1.54 (1.34-1.77) |  | 1.19 (0.999-1.42) | 1.14 (0.97-1.35) |
| Salivary IgA (ng/mL) |  |  |  |  |  |  |  |  |
| T1 |  |  |  |  |  |  |  |  |
| 10y | 97 | 36.0 (27.1-48.0) |  | 71 | 27.4 (20.5-36.7) |  | 1.31 (0.87-1.98) | 1.31 (0.88-1.95) |
| 12y | 112 | 66.8 (52.3-85.3) |  | 132 | 44.5 (36.1-54.9) |  | **1.50 (1.09-2.06)** | **1.48 (1.08-2.02)** |
| 15y | 118 | 64.1 (49.9-82.4) |  | 118 | 51.0 (40.3-64.5) |  | 1.26 (0.90-1.77) | 1.26 (0.90-1.76) |
| Overall | 327 | 54.8 (47.1-63.7) |  | 321 | 42.0 (36.6-48.3) |  | **1.36 (1.11-1.66)** | **1.36 (1.11-1.66)** |
| T2 |  |  |  |  |  |  |  |  |
| 10y | 93 | 10.6 (8.60-13.2) |  | 68 | 9.41 (7.60-11.7) |  | 1.13 (0.84-1.53) | 1.11 (0.83-1.50) |
| 12y | 101 | 15.9 (12.5-20.3) |  | 124 | 16.3 (13.2-20.0) |  | 0.98 (0.72-1.34) | 0.97 (0.72-1.32) |
| 15y | 114 | 18.3 (14.6-23.0) |  | 113 | 18.1 (14.5-22.6) |  | 1.01 (0.74-1.39) | 1.03 (0.76-1.39) |
| Overall | 308 | 14.8 (13.0-17.0) |  | 305 | 15.0 (13.2-17.0) |  | 1.03 (0.86-1.24) | 1.02 (0.86-1.22) |

Abbreviations: MenC, meningococcal serogroup C; IgA, immunoglobulin A; GMC, geometric mean concentration; CI, confidence interval; T0, before vaccination; T1, 1 month after vaccination; T2, 1 year after vaccination.

*Number of girls (F) and boys (M) excluded from the analysis due to missing IgA at T0: serum T1 10y: 4F, 1M; serum T1 12y: 2F, 1M; serum T1 15y: 4F, 2M; serum T1 overall: 10F, 4M; serum T2 10y: 3F, 1M; serum T2 12y: 2F, 1M; serum T2 15y: 4F, 2M; serum T2 overall: 9F, 4M; saliva T1 10y: 2F; saliva T1 12y: 3M; saliva T1 overall: 2F, 3M ; saliva T2 10y: 2F; saliva T2 12y: 2M ; saliva T2 overall: 2F, 2M.

**Supplementary Table 3. Geometric mean serum IgG concentrations and geometric mean concentration ratios for girls versus boys for tetanus toxoid (TT)-specific serum IgG at 1 month and 1 year following MenC-TT booster vaccination.**

|  | Girls | |  | Boys | |  | GMC ratio (95% CI) | |
| --- | --- | --- | --- | --- | --- | --- | --- | --- |
|  | n | GMC (95% CI) |  | n | GMC (95% CI) |  | adjusted for age group | adjusted for age group and IgA at T0 |
| TT-specific serum IgG (µg/mL) |  |  |  |  |  |  |  |  |
| T1 |  |  |  |  |  |  |  |  |
| 10y | 51 | 6.74 (5.65-8.04) |  | 37 | 8.43 (6.74-10.54) |  | 0.80 (0.61-1.05) | 0.93 (0.79-1.09) |
| 12y | 43 | 4.49 (3.61-5.58) |  | 46 | 4.31 (3.41-5.44) |  | 1.04 (0.77-1.42) | 1.15 (0.93-1.42) |
| 15y | 40 | 2.80 (2.21-3.70) |  | 45 | 2.94 (2.27-3.80) |  | 0.95 (0.66-1.37) | 0.89 (0.66-1.19) |
| Overall | 134 | 4.55 (3.96-5.23) |  | 128 | 4.57 (3.92-5.34) |  | 0.93 (0.77-1.11) | 0.98 (0.86-1.11) |
| T2 |  |  |  |  |  |  |  |  |
| 10y | 49 | 3.16 (2.66-3.75) |  | 36 | 4.39 (3.40-5.68) |  | **0.72 (0.54-0.96)** | 0.92 (0.77-1.09) |
| 12y | 43 | 1.75 (1.40-2.18) |  | 46 | 2.06 (1.62-2.62) |  | 0.85 (0.62-1.16) | 0.99 (0.88-1.12) |
| 15y | 40 | 1.16 (0.92-1.46) |  | 43 | 1.14 (0.86-1.51) |  | 1.02 (0.71-1.45) | 0.93 (0.74-1.17) |
| Overall | 132 | 1.92 (1.67-2.20) |  | 125 | 2.09 (1.76-2.49) |  | 0.85 (0.71-1.03) | 0.94 (0.85-1.04) |

Abbreviations: TT, tetanus toxoid; MenC, meningococcal serogroup C; IgG, immunoglobulin G; GMC, geometric mean concentration; CI, confidence interval; T0, before vaccination; T1, 1 month after vaccination; T2, 1 year after vaccination. No missings at T0.
